# iCollections – Digitising the British and Irish Butterflies in the Natural History Museum, London

**DOI:** 10.3897/BDJ.4.e9559

**Published:** 2016-09-13

**Authors:** Gordon Paterson, Sara Albuquerque, Vladimir Blagoderov, Stephen Brooks, Steve Cafferty, Elisa Cane, Victoria Carter, John Chainey, Robyn Crowther, Lyndsey Douglas, Joanna Durant, Liz Duffell, Adrian Hine, Martin Honey, Blanca Huertas, Theresa Howard, Rob Huxley, Ian Kitching, Sophie Ledger, Caitlin McLaughlin, Geoff Martin, Gerardo Mazzetta, Malcolm Penn, Jasmin Perera, Mike Sadka, Elisabetta Scialabba, Angela Self, Darrell J. Siebert, Chris Sleep, Flavia Toloni, Peter Wing

**Affiliations:** ‡ Natural History Museum, London, United Kingdom

**Keywords:** Collection digitisation, butterflies, British Isles, Ireland, mass digitsation, collections

## Abstract

**Background:**

The Natural History Museum, London (NHMUK) has embarked on an ambitious programme to digitise its collections . The first phase of this programme has been to undertake a series of pilot projects that will develop the necessary workflows and infrastructure development needed to support mass digitisation of very large scientific collections. This paper presents the results of one of the pilot projects – iCollections. This project digitised all the lepidopteran specimens usually considered as butterflies, 181,545 specimens representing 89 species from the British Isles and Ireland. The data digitised includes, species name, georeferenced location, collector and collection date - the what, where, who and when of specimen data. In addition, a digital image of each specimen was taken. This paper explains the way the data were obtained and the background to the collections which made up the project.

**New information:**

Specimen-level data associated with British and Irish butterfly specimens have not been available before and the iCollections project has released this valuable resource through the NHM data portal.

## Introduction

The Natural History Museum, London (NHMUK) has embarked on an ambitious programme to digitise its collections, some 80 million specimens (see http://www.nhm.ac.uk/our-science/our-work/digital-museum.html for background and details). The iCollections project was developed as part of this programme with the aim of developing the necessary data pipelines and digitisation workflows to undertake a mass digitisation project. In addition to the aim of digitising a large collection, iCollections was also established to test what systems would have to be developed and whether the existing infrastructure would be able to deal with relatively large volumes of data in a timely and secure way. This paper provides some of the background on how the British and Irish butterfly collections were digitised and the dataset created.

The data collected has been used by Brooks et al. (in press, Ecography) to examine the phenology of British butterflies between 1880 and 1980.

## General description

### Additional information

**Background to the iCollections project**

The NHMUK's British and Irish Lepidoptera collection is a large, comprehensive collection of British and Irish Lepidoptera, containing a wealth of material of both scientific and historic importance. The British and Irish specimens are separated from the main collection, making it easier to undertake a project of sufficient size to test various digitisation workflows. The discrete nature of the collection also made pre-digitisation preparation easier.

A number of factors were assessed that highlighted this collection as being an appropriate vehicle to develop and test mass digitization workflows of the NHMUK collections. Firstly, the project needed a coherent collection that would provide sufficient numbers of specimens to establish, test and develop suitable mass digitisation pipelines. Secondly, the collection needed to be of sufficient size to make an impact and to deliver a large volume of data. Thirdly, the project needed to be scientific and culturally coherent and credible – our entire British and Irish collections are of interest to scientists, conservationists and the general public. Fourthly, data produced could be used to address wider issues. For example have species ranges, flight times or morphology changed through time? Are they affected by climate change? What have we lost and what have we gained in terms of species? What species have been recorded in my area? Finally, from a collections management perspective – what do we have, how many, and where are the gaps?

A pilot study of four species of British and Irish butterflies carried out by Brooks et al. 2014 had established that a relatively straightforward workflow could be developed to capture label information which could be used in research projects. Phenological data could be gleaned from the collection/emergence dates on the labels and used to study the effects of a changing climate and how butterflies responded by shifting their seasonality. The temporal span of the collections (mainly mid-19^th^ century to 1980s) provided a useful historical perspective often absent in studies relying on more recent records. A more comprehensive analyses of the data has been completed (Brooks et al. in press, Ecography).

**Figure 1.**
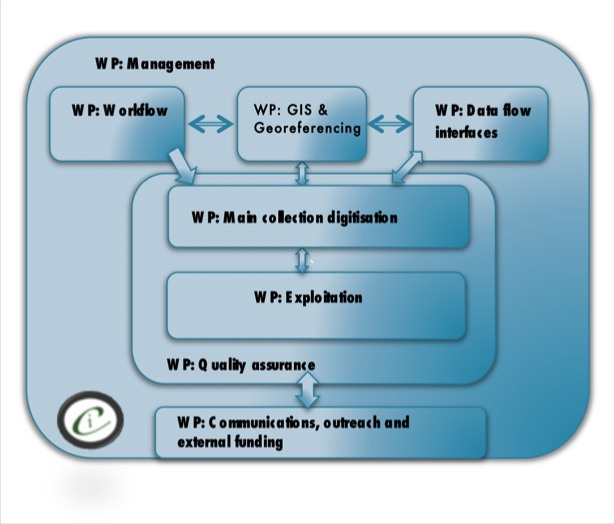
The work packages associated with the iCollections project

From early in the project development it was clear that expertise in a range of specialisms was going to be required for the project. Fig. 1 indicates the different specialist areas around which the project was organized. The specialisms employed in the project are also detailed below in the roles that the project team members performed. It is not an exaggeration to say that this project called on specialisms from across the Museum, ranging from HR/Personnel through IT to include research and collections management.

## Project description

### Title

iCollections: British and Irish Lepidoptera

### Personnel

***Roles in the project***

*Chair*: Gordon L J Paterson;

*Collections management*: Geoff Martin, Martin Honey, John Chainey, Blanca Huertas, Theresa Howard, Rob Huxley;

*QA/QC KE EMu*: Darrell Siebert;

*Workflows*: Vladimir Blagoderov, Steve Cafferty;

*Database/interfaces*: Adrian Hine, , Mike Sadka;

*Data operations and Automated processing of images*: Chris Sleep; *Research*: Steve Brooks, Ian Kitching;

*Digitisation Team*: Sara Albuquerque, Elisa Cane, Robyn Crowther, Lyndsey, Douglas, Joanna Durant, Sophie Ledger, Gerardo Mazzetta, Jasmin Perera, Elisabetta Scialabba, Flavia Toloni, Peter Wing;

*Georeferencing/GIS*: Malcolm Penn, Caitlin McLaughlin, Liz Duffell;

*Administration and project management*: Victoria Carter.

### Design description

***Digitisation process***

The digitisation process has three stages. The first is imaging. Specimens are photographed in a unit tray specially constructed to allow the specimens to be imaged together with their labels.

**Figure 2.**
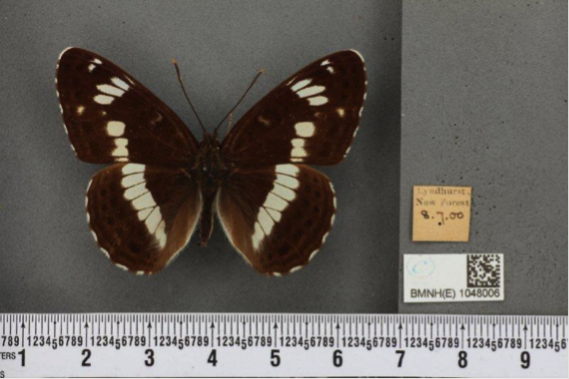
Image of a specimen in a unit tray ready to be imaged as part of the digitisation process.

Fig. 2 shows a specimen in a unit tray ready to be imaged. The label on the right is on an elevated section. The specimen is pinned so that it is approximately at the same height as the labels to avoid the need to refocus on different elements in the tray. Software is used to isolate and copy the image of the labels and this new image is made available for transcription (see below for details).

***File handling and storage***

The image files were placed into a shared folder structure organised to represent physical storage location and taxonomy. Once ready, a file system crawling process was invoked to carry out the initial steps: automatically cropping the label section, identifying barcodes and seeding the initial metadata to a database ready to support the transcription step. The files and associated data were then available to be ingested into the Museum’s collection management system.

**Figure 3.**
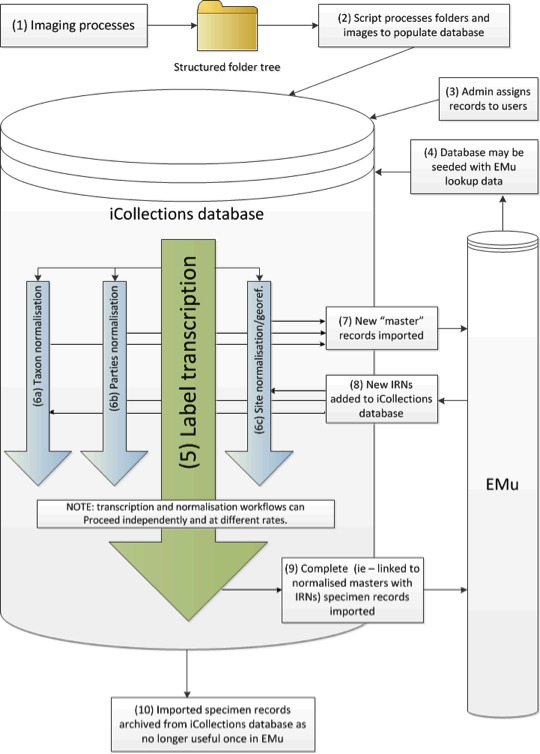
Workflow associated with the iCollections digitisation project.

The folder structure was organised to minimise the amount of manual entry needed during image capture, which in turn helps to maintain the rate at which the imaging can take place, and reduce the potential for initial capture errors. Fig. 3 shows the data workflow for the iCollections project.

The label information selected to be transcribed was atomised into different fields of a purpose designed MS Access™ transcription interface (the files were in a MS SQL Server™ database), focusing mainly on the ‘what’ (fields for taxonomy and primary type status, if any), ‘when’ (fields for date of collection and/or emergence), ‘where’ (site field), and ‘who’ (fields for collector and registration event). This information was transcribed by the digitisers without personal interpretation or conjecture. The interface allows the ingestion of multiple images when the information to be transcribed is placed on both sides of the labels; a visual cue warns the digitiser when this is the case.

**Figure 4.**
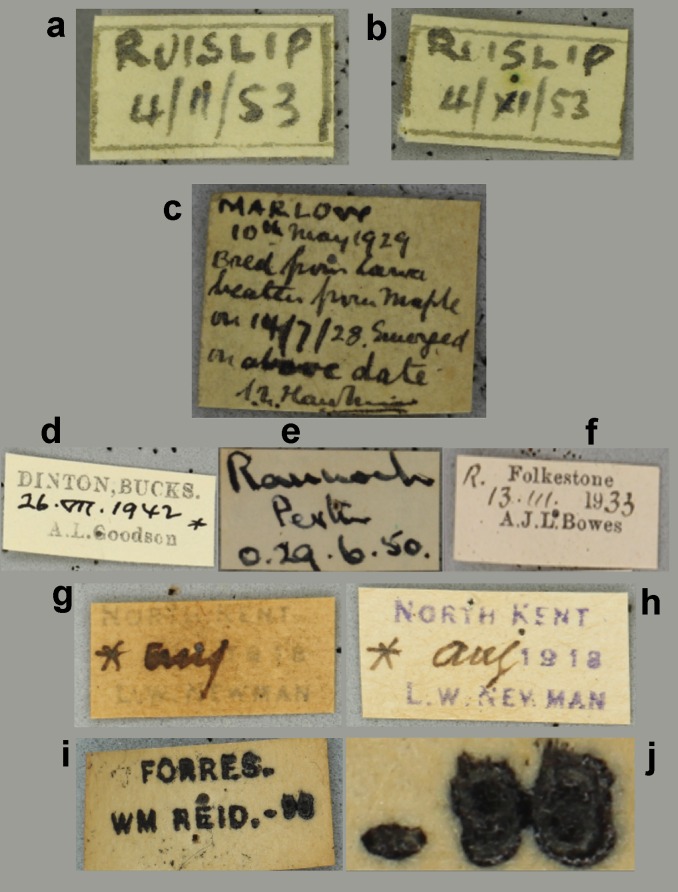
The label problem. a) and b) Transcription of ambiguous dates as exemplified by two handwritten labels from the locality (town) of Ruislip pertaining to different specimens; the middle number (month) on the date from the left label could be interpreted as February or November, depending on whether the number was written as a Roman numeral or not. The label on the right (b) has been handwritten by the same person and shows unequivocally that the middle number is indeed a Roman numeral but this finding also suggests that the collector also wrote 11 in the other label. c), d), e) and f) Transcription of emergence and collection dates. (c) Collection and emergence dates clearly stated on the handwritten label. (d) Emergence date inferred by known symbols (e.g., crosses and asterisks) and abbreviations or initials (e.g., ‘*B.*’ standing for ‘Bred’, ‘*l.*’ standing for ‘larva’), (e) ‘*o*’ standing for ‘ova’, or (f) ‘*R.*’ standing for ‘reared’. g) and h) Transcription of faded information from (g) barely legible label produced with a mimeograph; (h) A similar type of label in a better conservation state. i) and j) Transcription of a barely legible, smudged number (corresponding to the year that the specimen was collected) from a printed label was easily performed by zooming in its corresponding image, as shown in the enlarged detail on the right. It became apparent that the stamped number was 96, and consequently the collection year was assumed to be 1896 (century inferred based on known period of activity for W.M. Reid).

Locality and collector names can be written in many, very different styles or using slightly different spellings (variants), which were first transcribed *verbatim* and then harmonised (i.e., converted into a standardised format) at a later stage during georeferencing and migration into the NHM’s Collections Management System (KE EMu™). Moreover, dates (either Fig. 4 collection or emergence data) on labels were written in different formats and often qualified as such by the use of symbols or abbreviations, so a certain amount of *a priori* or on-the-job knowledge was necessary to correctly transcribe this information. The interface provided several ‘follow up’ options relevant to a field when data are illegible, unknown, or uncertain (e.g., ‘Label comments’ and ‘Admin comments’ fields); this type of data was scrutinised and interpreted at a later stage by experienced museum curators and researchers.

**Figure 5.**
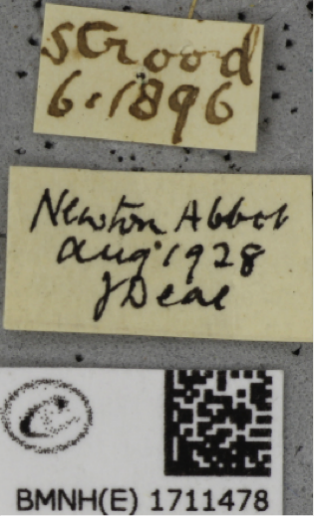
Erroneous labels

Examples of the range of problems encountered when transcribing specimen labels is given in Fig. 4. An addition problem is that the information on the labels can sometimes be erroneous (e.g., conflicting information on different labels attached to the same specimen) or misleading, requiring further scrutiny by museum curators. Fig. 4 g shows the transcription of faded information from a barely legible label produced with a mimeograph and Fig. 4 h shows a similar type of label in a better conservation state. Fig. 4 (i, j) show labels which are either only partially legible or completely smudged and unclear, e.g. the number in this case is smudged (corresponding to the year that the specimen was collected) but was easily deciphered by zooming in on its corresponding image, as shown in the enlarged detail Fig. 4 (j). It became apparent that the stamped number was 96, and consequently the collection year was assumed to be 1896 (the century was inferred, based on known period of activity for W.M. Reid). Fig. 5 shows an example of erroneous and misleading label information, in this case conflicting information on collecting localities and dates. Resolving such conflicts relied on the knowledge of the curator about the collector in question and whether they had collected other specimens from the same locality or at the same time. These other records can help decide which of the localities was most likely. But it was not always possible to resolve the conflict for some records.

​***Developing interfaces for data capture***.

**Figure 6.**
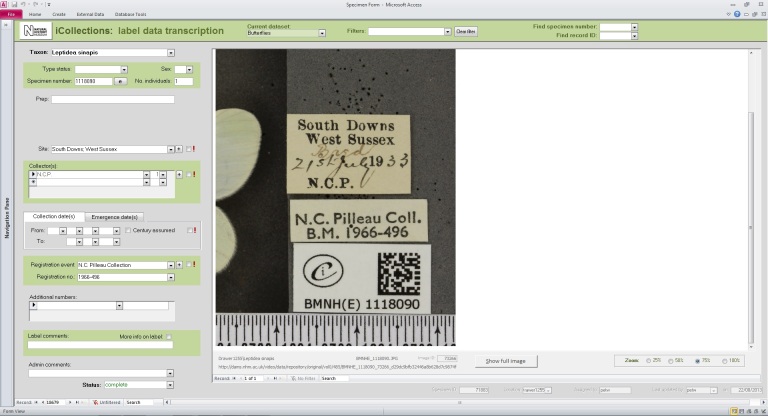
Label transcription interface.

**Figure 7.**
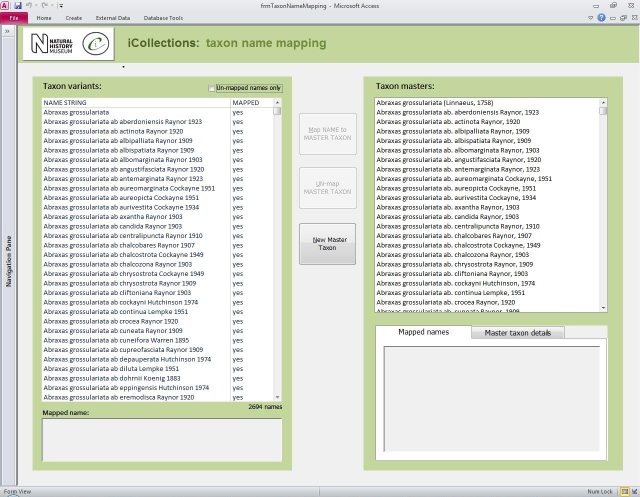
Taxon standardisation inferface.

**Figure 8.**
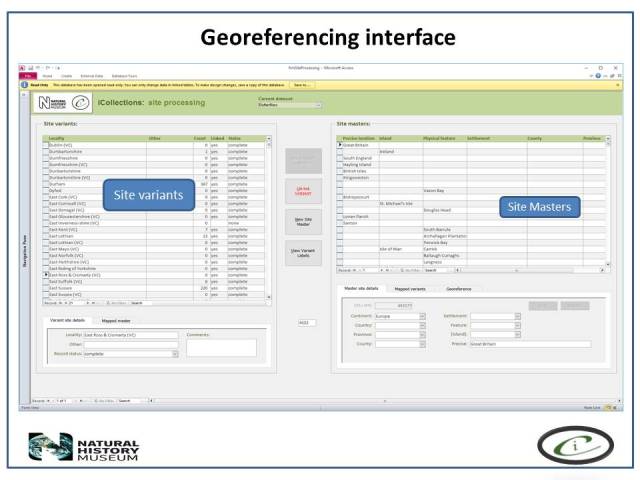
Georeferencing site variant harmonisation interface.

**Figure 9.**
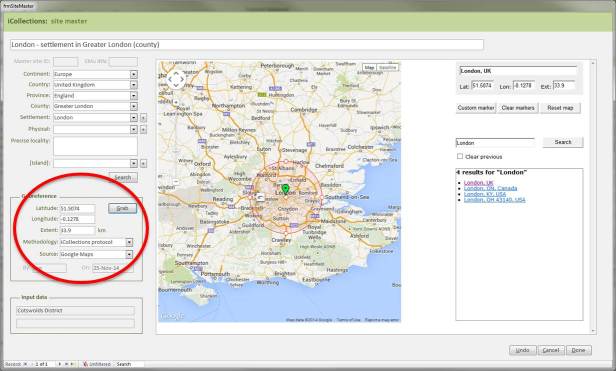
Georeferencing interface

**Transcription interface.** To capture the data on the labels a series of interfaces for transcription were developed. The transcription interface operates against a normalised database, which reduces data capture effort (keyed values are available to all transcribers) and transcription errors (once keyed, data values exist only once in the system, and can be selected as appropriate rather than being re-keyed). Three types of records were selected and had look-up lists developed: taxa, sites (and georeferencing) and Parties–collector and/or donor (Figs 6, 7, 8, 9).

The interfaces were designed using MS Access 2010 ™. This software was selected for a number of reasons, in particular its flexibility. MS Access 2010 ™ is an excellent front end for MS SQL Server ™ and is a a recognised industry standard with dedicated record handling environment, providing many conveniences and thereby facilitating rapid development.

In developing the interfaces great care was taken to experiment with various facets of the interface, for example, changing the colours around the interface fields so that they were more easily recognised and separated. This phase of developing and trialling the interfaces relied on good communication between the digitisation team and the interface developers. It was clear that small changes could achieve large efficiency gains by making the interface more intuitive to use and organising the fields in ways that improve the flow (see) .

***Georeferencing***

It is important that the entire georeferencing process follows a set of guidelines that are designed to reduce georeferencing error and increase repeatability. The NHM georeferencing guidelines provide clear instructions on how to approach and georeference different spatial locations, e.g. mountains, rivers, when to use "near” and the application of standard abbreviations etc., They allowed the team to maintain geographical standards based on WGS 1984 Decimal Degrees and importantly, ensure consistency was carried forward. The guidelines are continuously updated as project digitise other collections across the NHM, so that they provide a clear set of instructions of how to georeference specific localities and their extents, thereby, providing a geographical standard, based on our NHM data and best practise from other similar organisations.

Finally, the whole process of georeferencing at the NHM was designed to clean the data, normalisation/harmonisation and map site variants to unambiguous master records reducing the number of site records that need to be imported into the collections management data system, standardise while following both NHM and geographical standards (e.g. ISO 2015). This process of reducing duplication and error to produce a site master reduced the final tally of iCollection sites georeferenced by approximately 50%.

**Georeferencing process**

The Georeferencing process was split into five broad phases.

- **First phase**. During this stage only sites with at least 5 specimens collected were georeferenced, (this enables sites with many specimens to be georeferenced quickly, these can account for 60-70% of a collection).
- **Second phase**. The team split the remaining data into two parts A-M and N–Z, and no more than 15 minutes was spent on each site trying to georeference the data.
- **Third phase**. The remaining data were then checked by the senior georeferencer and any queries addressed and the data were then further investigated by the georeferencers and the specific curator /researcher, who provide help on any specific problem locations.
- **Fourth phase**. The remaining data were checked that a georeference was not obtainable and these were then noted as a un-georeferenceable in the dataset. The dataset was then checked for accuracy by taking a random 100 sites from the data and compare results.
- **Fifth phase**. Data were then exported and ingested into KeEMU ™ by the NHM database team.

***Digitisation timings and costs***

The timings to prepare the specimens, image and transcribe label information varied from 0.52 to 4.52 minutes with the modal figure around 2.4 minutes. Estimating the cost of the digitisation process depends on what is included. For example, it is possible to calculate the costs based on the funds needed to hire new staff and buy equipment But it is also possible to calculate the cost per specimen based on an assessment of all the hidden costs such as the time spent on the project by existing staff. This can lead to different estimates of the project cost and ultimately the cost of digitising each specimen. The cost per specimen based only on the hire of new staff and the purchase of new equipment was £1.17 (based on the 2015 NHM salary scales). However, if one also takes into account the time spent by in-house staff to support the project, the expense of refurbishing accommodation, hire of new staff, equipment, etc., then the true cost per specimen is nearer £2.02. It is important to be clear about what costs are being assessed and included when the cost per specimen is given.

## Geographic coverage

### Description

**Figure 10.**
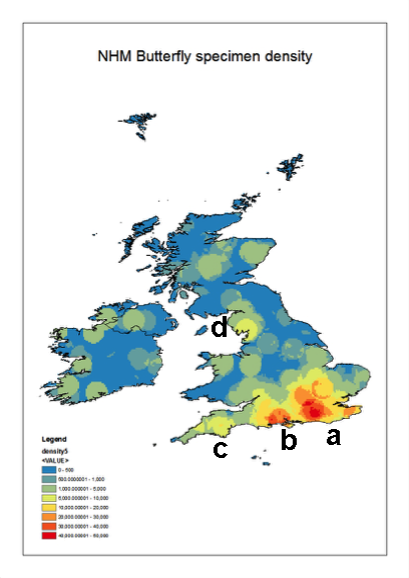
Map giving the specimen distribution within the collections. a- Home Counties around London; b – New Forest; c – Torbay region and d) Lake District. The latter two localities became popular holiday destinations during the late 1800s and early 1900s.

The collections cover most of the British Isles and Ireland. However, there are distinct ‘hotspots’ particularly along the south coast of England. These reflect the areas frequented by the main collectors represented in the collections. The densest collecting area is around London and the Home Counties (Fig. 10), which would have been easy to access using public transport particularly as the rail network developed at the end of the 19th and early 20^Th^ centuries. There is also a secondary focus (b on the map) west of the main area which is the New Forest region.

## Taxonomic coverage

### Description

**Figure 11.**
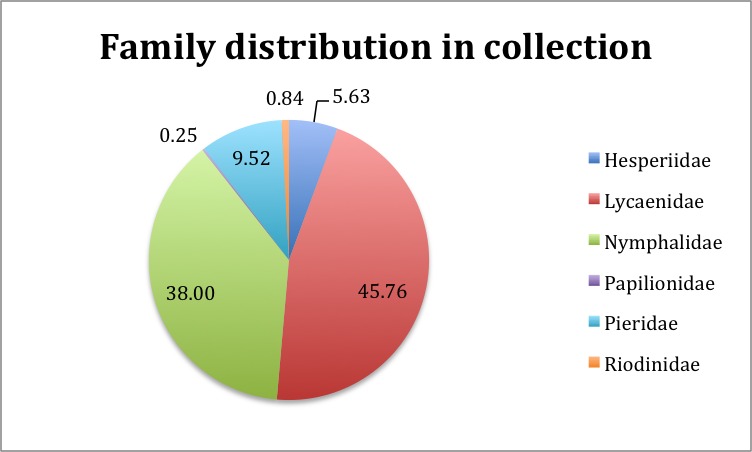
Chart showing the percentage of specimens belonging to the different families within the British and Irish Lepidoptera collection.

The Project focused on Lepidoptera from the families Lycaenidae, Nymphalidae, Pieridae, Riodinidae, Hesperiidae, Papilionidae collectively referred to in the project as butterflies. Fig. 11 shows the percentage breakdown of the different families in the collection. Table 1 (Suppl. material 1) lists the species recoded and the numbers of individuals of each species in the NHMUK collections. Taxonomy follows Agassiz et al. 2013. All 89 resident, migrant and accidentally introduced species (which include 142 subspecies and 2232 infrasubspecies) were digitised and databased provides the taxonomic coverage and the abundance of the different species and subspecies in the collection. British lepidopterists became interested, perhaps obsessed with variants and aberrations of species. In digitising the collections we decided to try to capture the published variants/ aberrations information as this was likely to be of general interest.

The British and Irish butterfly collections was built up over many years through the purchase of smaller collections and individual collecting. Salmon et al. 2000 provides a biography of the main collectors in the British Isles and history of butterfly collecting over the past two centuries. Another feature of the collection is the large number of bred specimens. Many of these would have been bred to obtain perfect specimens for the cabinet or a series to show the variation within the species concerned, or to try to obtain a new aberration. Newman 1967 provides an interesting account of this aspect of butterfly collecting.

## Temporal coverage

### Notes

**Figure 12.**
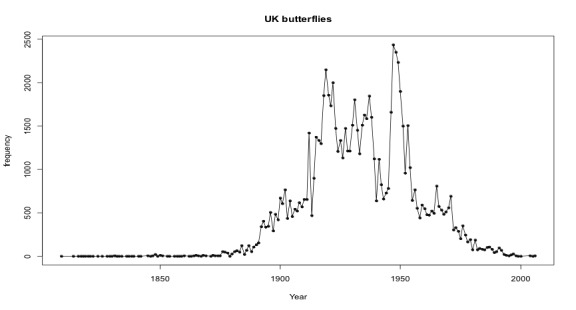
Growth of collections with time.

The NHMUK British and Irish collections span nearly two centuries. The collecting of butterflies became a popular pastime for amateur and professional collectors alike with the main period of collecting spanning the turn of the 20^th^ century until the mid 1950s (Fig. 12). The decline reflects the change in attitudes to collecting specimens with a greater emphasis on photography and observation in recent years but also a change in Museum policy on collection acquisition.

**Figure 13.**
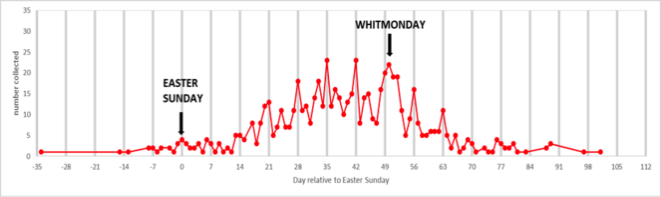
The graph indicates the when during the year specimens of the Orange Tip (*Anthocharis cardamines*) butterfly were collected. The x- axis shows the day of the year while the y-axis indicates the number of specimens collected.

Fig. 13 illustrates the link between the time of the year and day of the week and the numbers of butterflies collected, in this case the Orange Tip (*Anthocharis cardamines*). Further analysis of the temporal data suggests changes in the social conditions in the British Isles. Fig. 13 shows that the main collecting effort (peaks) happened at weekend days – Saturday or Sunday - and bank holidays. Butterfly collecting begins around springtime reaching a peak over the spring months with the late May Bank Holiday weekend particularly prominent (Whit Monday). This temporal pattern suggests that there was an increase in leisure time coupled with sufficient disposable income, for at least some of the population, to be able to indulge in hobbies like butterfly collecting. Some preliminary assessment of the collections also suggested that the geographic spread of the specimens collected over the main collecting period follows the development of the rail network and increasingly cheaper fares.

## Collection data

### Collection name

iCollections British and Irish butterflies

### Collection identifier

Gordon L J Paterson; Sar﻿a Albuquerque; Vladimir Blagoderov; Steve Brooks, et al. (2016). Dataset: iCollections. https://doi.org/10.5519/0038559

### Curatorial unit

Three large collections bequeat﻿hed to the NHMUK comprise the core of the British and Irish Lepidoptera collection, the Rothschild, Cockayne and Kettlewell collections.

## Usage rights

### Use license

Creative Commons Public Domain Waiver (CC-Zero)

## Data resources

### Data package title

Gordon L J Paterson; Sara Albuquerque; Vladimir Blagoderov; Steve Brooks, et al. (2016). Dataset: iCollections. https://doi.org/10.5519/0038559

### Number of data sets

1

### Data set 1.

#### Data set name

iCollections British and Irish butterflies

#### Number of columns

15

#### Download URL

https://doi.org/10.5519/0038559

#### Description

Specimen data of British and Irish butterflies giving identification (including aberrations and forms), locality, collector and date of collector (where known).

**Data set 1.** 

Column label	Column description
GBIF	GBIF quality status
Catalogue	NHMUK catalogue number
Scientific name	Genus and species name
Author	Author name and date
Type status	Type status
Locality	Geographic locality
Country	Country
Records	Collector
Collection	Which NHMUK collection specimen record is from
Class	Taxonomic class
Family	Lepidopteran family
Genus	Genus
Species	Species
Subspecies	Subspecies
Project	Project title

## Supplementary Material

Supplementary material 1 List of butterfly species in the NHMUK British and Irish Collections Data type: Recods of the numbers of individuals of each British and Irish species
Brief description: CSV file alternative to Table 1
File: oo_102024.csv Paterson, G.L.J. et al

## Figures and Tables

**Table 1.** Table 1. Species in the NHMUK British and Irish butterfly collections together with counts of the numbers of specimens of each species.

**Agassiz number**	**Scientific name**	**Count**	**Notes**
56.002	*Iphiclides podalirius* (Linnaeus, 1758)	1	
56.003	*Papilio machaon* Linnaeus, 1758	810	
56.0031	*Papilio xuthus* Linnaeus, 1767	1	UK data
57.001	*Erynnis tages* (Linnaeus, 1758)	1215	
57.0012	*Pyrgus armoricanus* (Oberthür, 1910)	3	UK data
57.002	*Pyrgus malvae* (Linnaeus, 1758)	1673	
57.0021	*Pyrgus serratulae* (Rambur, 1839)	1	UK data
57.004	*Carterocephalus palaemon* (Pallas, 1771)	950	
57.005	*Thymelicus lineola* (Ochsenheimer, 1808)	854	
57.006	*Thymelicus sylvestris* (Poda, 1761)	1250	
57.007	*Thymelicus acteon* (Rottemberg, 1775)	711	
57.008	*Hesperia comma* (Linnaeus, 1758)	875	
57.009	*Ochlodes sylvanus* (Esper, 1779)	1249	
58.001	Leptidea sinapis (Linnaeus, 1758)	1763	Irish specimens not dissected. Recently split into two species the collection has yet to be reidentified.
58.002	*Leptidea juvernica* Williams, 1946	4	
58.003	*Anthocharis cardamines* (Linnaeus, 1758)	2866	
58.005	*Aporia crataegi* (Linnaeus, 1758)	451	
58.006	*Pieris brassicae* (Linnaeus, 1758)	2281	
58.007	*Pieris rapae* (Linnaeus, 1758)	2413	
58.008	*Pieris napi* (Linnaeus, 1758)	8164	
58.009	*Pontia daplidice* (Linnaeus, 1758)	160	
58.01	*Colias croceus* (Fourcroy, 1785)	2749	
58.011	*Colias hyale* (Linnaeus, 1758)	662	
58.012	*Colias alfacariensis* Berger, 1948	65	
58.0121	*Colias phicomone* (Esper, 1780)	1	UK data
58.013	*Gonepteryx rhamni* (Linnaeus, 1758)	884	
58.014	*Gonepteryx cleopatra* (Linnaeus, 1767)	4	4 UK data
59.001	*Danaus plexippus* (Linnaeus, 1758)	24	
59.002	*Lasiommata megera* (Linnaeus, 1767)	1907	
59.003	*Pararge aegeria* (Linnaeus, 1758)	3037	
59.004	*Coenonympha tullia* (Müller, 1764)	5293	
59.005	*Coenonympha pamphilus* (Linnaeus, 1758)	4008	
59.006	*Erebia ligea* (Linnaeus, 1758)	5	3 UK data, 2 No data
59.007	*Erebia epiphron* (Knoch, 1783)	1422	
59.008	*Erebia aethiops* (Esper, 1777)	1734	
59.009	*Aphantopus hyperantus* (Linnaeus, 1758)	3686	
59.01	*Maniola jurtina* (Linnaeus, 1758)	7118	
59.011	*Pyronia tithonus* (Linnaeus, 1771)	3016	
59.012	*Melanargia galathea* (Linnaeus, 1758)	2706	
59.013	*Hipparchia semele* (Linnaeus, 1758)	2878	
59.0131	*Hipparchia fagi* (Scopoli, 1763)	1	UK data
59.0132	*Arethusana arethusa* ([Denis & Schiffermüller], 1775)	1	UK data
59.0137	*Dryas julia* (Fabricius, 1775)	1	UK data
59.0138	*Heliconius charithonia* (Linnaeus, 1767)	1	UK data
59.014	*Boloria euphrosyne* (Linnaeus, 1758)	2697	
59.015	*Boloria selene* ([Denis & Schiffermüller], 1775)	3097	
59.0151	*Boloria dia* (Linnaeus,1767)	1	No data
59.016	*Issoria lathonia* (Linnaeus, 1758)	119	
59.017	*Argynnis paphia* (Linnaeus, 1758)	2164	
59.019	*Argynnis aglaja* (Linnaeus, 1758)	1882	
59.02	*Argynnis adippe* ([Denis & Schiffermüller], 1775)	1463	
59.0201	*Argynnis niobe* (Linnaeus, 1758)	4	3 UK, 1 no data; Agassiz number B36
59.021	*Limenitis camilla* (Linnaeus, 1764)	1169	
59.022	*Apatura iris* (Linnaeus, 1758)	566	
59.0221	*Colobura dirce* (Linnaeus, 1758)	1	UK data
59.0221	*Parthenos sylvia* (Cramer, 1775)	1	UK data
59.023	*Vanessa atalanta* (Linnaeus, 1758)	1268	
59.024	*Vanessa cardui* (Linnaeus, 1758)	957	
59.025	*Vanessa virginiensis* (Drury, 1773)	2	
59.0251	*Vanessa indica* (Herbst, 1794)	1	UK data
59.0252	*Hypanartia lethe* (Fabricius 1793)	1	UK data
59.026	*Aglais io* (Linnaeus, 1758)	1280	
59.027	*Aglais urticae* (Linnaeus, 1758)	3604	
59.028	*Nymphalis antiopa* (Linnaeus, 1758)	134	
59.029	*Nymphalis polychloros* (Linnaeus, 1758)	776	
59.03	*Nymphalis xanthomelas* (Esper, 1781)	1	
59.031	*Polygonia c-album* (Linnaeus, 1758)	2044	
59.032	*Araschnia levana* (Linnaeus, 1758)	10	UK data
59.0321	*Junonia oenone* (Linnaeus, 1758)	1	UK data
59.033	*Euphydryas aurinia* (Rottemberg, 1775)	6423	
59.034	*Melitaea cinxia* (Linnaeus, 1758)	1870	
59.036	*Melitaea athalia* (Rottemberg, 1775)	2253	
60.001	*Hamearis lucina* (Linnaeus, 1758)	1710	
61.001	*Lycaena phlaeas* (Linnaeus, 1761)	5996	
61.002	*Lycaena dispar* (Haworth, 1802)	1098	345 UK
61.0021	*Lycaena hippothoe* (Linnaeus, 1761)	7	6 No data, 1 UK data; Agassiz number B40
61.0022	*Lycaena virgaureae* (Linnaeus, 1758)	8	3 UK, 5 no data; Agassiz number B39
61.003	*Thecla betulae* (Linnaeus, 1758)	1199	
61.004	*Favonius quercus* (Linnaeus, 1758)	1197	
61.005	*Callophrys rubi* (Linnaeus, 1758)	1425	
61.006	*Satyrium w-album* (Knoch, 1782)	990	
61.007	*Satyrium pruni* (Linnaeus, 1758)	893	
61.0071	*Deudorix antalus* (Hopffer, 1855)	1	UK data, (2 specimens in original list)
61.0071	*Rapala schistacea* (Moore, 1881)	2	UK data
61.008	*Lampides boeticus* (Linnaeus, 1767)	34	
61.0081	*Cacyreus marshalli* Butler, 1898	3	
61.01	*Cupido minimus* (Fuessly, 1775)	1937	
61.011	*Cupido argiades* (Pallas, 1771)	4	2 No data, 2 UK
61.012	*Celastrina argiolus* (Linnaeus, 1758)	2298	
61.013	*Maculinea arion* (Linnaeus, 1758)	1827	
61.01305	*Maculinea alcon* ([Denis & Schiffermüller], 1775)	1	UK data
61.0131	*Glaucopsyche alexis* (Poda, 1761)	2	1 UK data, 1 No data
61.014	*Plebejus argus* (Linnaeus, 1758)	9094	
61.0141	*Aricia* Reichenbach, 1817	124	No locality data hence specimens could not be assigned to either *A. agestis* or *A. artaxerxes*
61.015	*Aricia agestis* ([Denis & Schiffermüller], 1775)	2512	
61.016	*Aricia artaxerxes* (Fabricius, 1793)	1836	
61.0161	*Aricia agestis × artaxerxes*	1637	Bred hybrids
61.017	*Cyaniris semiargus* (Rottemberg, 1775)	229	
61.018	*Polyommatus icarus* (Rottemberg, 1775)	12495	
61.0181	*Polyommatus dorylas* ([Denis & Schiffermüller], 1775)	2	2 UK data; Agassiz number B43
61.019	*Lysandra bellargus* (Rottemberg, 1775)	8592	
61.02	*Lysandra coridon* (Poda, 1761)	21702	
61.0201	*Lysandra coridon × bellargus*	3	Naturally occurring hybrids
